# Necrotizing pneumonia caused by methicillin‐resistant *Staphylococcus aureus*

**DOI:** 10.1002/ccr3.5619

**Published:** 2022-03-21

**Authors:** Toshiki Hiramatsu, Kazunori Tobino

**Affiliations:** ^1^ 13750 Department of Critical Care Medicine Iizuka Hospital Iizuka Japan; ^2^ 13750 Department of Respiratory Medicine Iizuka Hospital Iizuka Japan

**Keywords:** methicillin‐resistant *Staphylococcus aureus*, necrotizing pneumonia, vancomycin

## Abstract

We report a fatal case of methicillin‐resistant *Staphylococcus aureus* (MRSA)‐induced necrotizing pneumonia that was refractory to adequate vancomycin treatment (trough value, 13.1 µg/ml), drainage of hydropneumothorax, and veno‐arterial extracorporeal membrane oxygenation. Despite appropriate treatment, MRSA infection can cause rapidly progressive disease with a high‐case fatality rate.

## Graphical abstract

We report a fatal case of methicillin‐resistant *Staphylococcus aureus* (MRSA)‐induced necrotizing pneumonia that was refractory to adequate vancomycin treatment (trough value, 13.1 µg/ml), drainage of hydropneumothorax, and veno‐arterial extracorporeal membrane oxygenation. Despite appropriate treatment, MRSA infection can cause a rapidly progressive disease with a high‐case fatality rate.

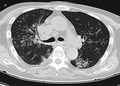

**What** **bacteria caused this pneumonia? Could prognosis have been predicted?**

**Response:**

## 1 CASE DISCUSSION

**FIGURE 1:**
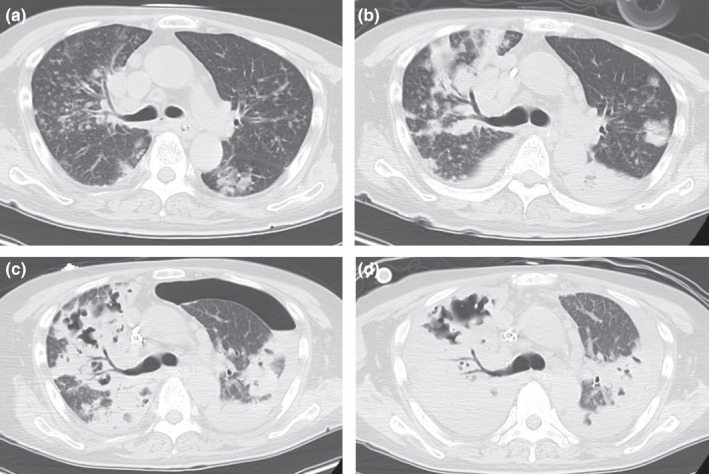
Computed tomography (CT) showing rapid progression of the necrotizing pneumonia caused by methicillin‐resistant *Staphylococcus aureus*, despite appropriate antibiotic therapy. (A) Plain CT showing bilateral multilobular lung nodules and bronchial wall thickening on Day 1. (B) Chest CT showing the expansion of bilateral consolidation on Day 3. (C) Chest CT showing necrotizing pneumonia with a left hydropneumothorax on Day 10. (D) Chest CT showing expansion of the cavity in the patient's left lung on Day 13

A 63‐year‐old man with diabetes mellitus and hypothyroidism presented with dyspnea for a couple of days following a two‐week history of cough and throat pain. On examination, he was febrile (38.2°C) with Glasgow Coma Scale of 14(E4V4M6), tachypneic (32 breaths/min), and hypotensive (blood pressure, 102/74 mmHg). He was intubated due to his shock state, admitted to the intensive care unit, and treated with intravenous vancomycin. Chest computed tomography (CT) revealed multiple centrilobular lung nodules and bronchial wall thickening bilaterally (Figure 1A). Vancomycin‐susceptible methicillin‐resistant *Staphylococcus aureus* (MRSA) was identified in blood and sputum cultures (vancomycin MIC =1 μg/ml). On Day 3, chest CT revealed expansion and consolidation within the right lung (Figure 1B). The vancomycin trough level was adequate (13.1 µg/ml). Veno‐arterial extracorporeal membrane oxygenation was initiated on Day 5. However, his clinical condition worsened. Chest CT on Day 10 (Figure 1C) showed worsening lung consolidation, multiple cavities, and left hydropneumothorax. He was diagnosed with necrotizing pneumonia, and a chest drain was placed in the left thoracic cavity. On Day 13, chest CT (Figure 1D) showed increased left lung cavitation. He died on Day 18. It is predicted that rapidly progressive destructive pneumonia based on initial CT findings was impossible. Despite appropriate treatment, MRSA‐induced necrotizing pneumonia can be rapidly progressive and fatal.^1^, ^2^

## CONFLICT OF INTEREST

None declared.

## AUTHOR CONTRIBUTIONS

Toshiki Hiramatsu involved in patient care and wrote the original manuscript. Kazunori Tobino involved in patient care, edited and revised the original manuscript.

## CONSENT

Appropriate written informed consent was obtained from the patient's brother for the publication of this case report and accompanying images in accordance with the journal's patient consent policy.

## Data Availability

All of the data that pertain to this report are available from the corresponding author upon reasonable request.

## References

[1] Chatha N , Fortin D , Bosma K . Management of necrotizing pneumoniae and pulmonary gangrene: a case series and review of the literature. Can Respir J. 2014;21(4):239‐245. 24791253 10.1155/2014/864159 PMC4173892

[2] del Carpio‐Orantes L . Necrotizing pneumonia or pulmonary gangrene. Community Acquir Infect. 2017;4:56‐58.

